# Strong, Long-Term Temporal Dynamics of an Ecological Network

**DOI:** 10.1371/journal.pone.0026455

**Published:** 2011-11-17

**Authors:** Jens M. Olesen, Constantí Stefanescu, Anna Traveset

**Affiliations:** 1 Institute of Bioscience, Aarhus University, Aarhus, Denmark; 2 The Butterfly Monitoring Scheme, Museu Granollers-Ciències Naturals, Barcelona, Spain; 3 Institut Mediterrani d'Estudis Avançats (Consejo Superior de Investigaciones Científicas-Universitat de les Illes Balears), Esporles, Mallorca, Spain; Centre National de la Recherche Scientifique, France

## Abstract

Nature is organized into complex, dynamical networks of species and their interactions, which may influence diversity and stability. However, network research is, generally, short-term and depict ecological networks as static structures only, devoid of any dynamics. This hampers our understanding of how nature responds to larger disturbances such as changes in climate. In order to remedy this we studied the long-term (12-yrs) dynamics of a flower-visitation network, consisting of flower-visiting butterflies and their nectar plants. Global network properties, i.e. numbers of species and links, as well as connectance, were temporally stable, whereas most species and links showed a strong temporal dynamics. However, species of butterflies and plants varied bimodally in their temporal persistance: Sporadic species, being present only 1–2(-5) years, and stable species, being present (9-)11–12 years, dominated the networks. Temporal persistence and linkage level of species, i.e. number of links to other species, made up two groups of species: Specialists with a highly variable temporal persistence, and temporally stable species with a highly variable linkage level. Turnover of links of specialists was driven by species turnover, whereas turnover of links among generalists took place through rewiring, i.e. by reshuffling existing interactions. However, in spite of this strong internal dynamics of species and links the network appeared overall stable. If this global stability-local instability phenomenon is general, it is a most astonishing feature of ecological networks.

## Introduction

Diversity, stability and dynamics of Mother Nature are highly influenced by the structuring of her species and their interactions. These are organized into hierarchical and heterogeneous networks of high complexity [1], [2] and they often reveal surprising, emergent, higher-order regularities, e.g. [3], [4]. However, almost all ecological network studies to date are snapshots of limited temporal extent [5], [6], i.e., network studies extending beyond a few years in scale lack any finer temporal resolution. This hampers our ability to tease apart “natural” network variation from variation caused by human-induced disturbances like climatic changes and invasion of aliens. Thus we need long-term, detailed temporal datasets of networks, e.g. [7]–[10], but these are rarely available. A few 2–4 year studies, however, reach somewhat congruent conclusions: overall or “globally”, ecological network structure seems steady over time, but “locally”, species and their connecting links show strong turnover, e.g. [11]–[13].

Across disciplines, networks of widely different nature reveal an astonishing generality in structure, e.g. a few, simple rules may generate similar patterns in both pollination networks and business networks [14]. Since we expect structure and dynamics to run in tandem, knowledge of structure and dynamical behaviour of pollination networks is of value in our efforts to understand other kinds of network as well.

We examined the long-term dynamics of a flower-visitation network between butterflies and their nectar plants by, in detail, locating topological “hot and cold spots” of high and low temporal dynamics, respectively. This internal, long-term temporal dynamics in empirical networks is virtually unknown.

## Materials and Methods

### Permissions and ethical issues

All data and species were sampled according to the Butterfly Monitoring Scheme, Museu Granollers-Ciències Naturals, Barcelona, to which CS is affiliated. The scheme gathers baseline information, and research applications, in general, have to be approved by the above institution. No specific permits were required to work at the study sites.

### Study sites

We made our observations at El Puig (UTM: 507851, 4674362), an open Mediterranean shrubland in NE Spain, ranging up to 1100 m a.s.l. In addition, we made the same data sampling at three other study sites nearby (SI 1).

### Data sampling

Our dataset had a resolution based on 30 weekly samplings per year over 12 years (March–September, 1996–2007). Here we only used the El Puig dataset. All butterflies within 2.5 m on each side of a 2,029 m long transect and 5 m in front of the recorder were counted [15], [16]. This census is part of the Catalan Butterfly Monitoring Scheme [www.catalanbms.org/]. We scored all interactions between species of flower-visiting butterflies and their nectar plants, and their frequency. Flower-visiting butterflies were only recorded, if they interacted with flowers, i.e. probed for nectar. A link was established if at least one individual of a butterfly species visited a flowering plant species for nectar. This fixed observation protocol throughout all 12 years kept sampling variability at a minimum. In addition, information about geographic range was gathered from www.tyllinen.eu/Butterflies/Butterflies.htm. ([17]; [16] uses the same dataset as our paper). In the beginning of the season, we spent one hour per week along the transect, and later in mid-season (July), we spent 2–3 hours weekly; the exact amount of time depended upon the number of butterflies seen.

### Data analysis

All data were arranged in annual, bipartite plant-butterfly interaction matrices. Using these 12 annual snapshots, we analyzed the temporal dynamics from year to year of each species and its links in relation to their topological position in the network. Specialization/generalization of a species was measured as its linkage level *L*, i.e. number of links to other species. It is a simple, crude, but fundamental local network descriptor. One key feature of many bipartite ecological networks is, that their link pattern is nested, i.e. links of specialized species are subsets of links of more generalized species [18]. To calculate level of nestedness *N* in the network, we used the program *ANINHADO* and its index *NODF* [19], [20]. Using data from our 12 annual networks and thus 11 annual transitions, we calculated the number of colonization and extinction events for each species and link. A colonization was the appearance of a species or a link in the network after ≥1 year of absence and *vice versa* for an extinction. If species and links did not colonize or go extinct they survived from one year to the next or remained absent. Extinction and colonization probabilities (*e* an *c*) for a species or a link were here defined as *e* = (no. extinctions during 12 yrs)/(no. extinctions during 12 yrs + no. survivals during 12 yrs) and *c* = (no. colonizations during 12 yrs)/(no. colonizations during 12 yrs + no. survivals during 12 yrs). Mean annual turnover rate of a species or a link was *t* = (*e*+*c*)/2.

## Results

**Figure 1:**
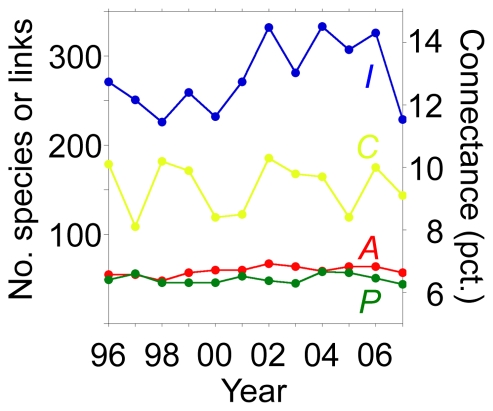
Temporal variation in basic butterfly–nectar plant network characteristcs. Numbers of butterfly species (*A*), plant species (*P*), links (*I*), and connectance (*C*) during 12 years of study.

Pooling data over all years, 87 butterfly species (*A*) made 1,134 nectar-visit interactions (*I*) to 109 flowering plant species (*P*) producing a connectance *C* = 100*I*/(*AP*) of the network of 12.0%. This plant-butterfly flower-visitation network was a subset of the total flower-visitation network, which also encompassed other insect groups and additional plants not visited by butterflies. However, on average, only 59.3±5.4 (mean ± SD) butterfly species (68% of *A*), 49.8±5.0 plant species (46% of *P*), and 276.3±39.6 links (only 24% of *I*), were observed in the network each year. Thus average annual *C* became 9.4%. The temporal stability of these parameters may be described by their levels of long-term trends and short-term fluctuations. The annual numbers of plant species and links, as well as connectance did not vary among years (simple linear regression: *F*_Plants_ = 0.29, *p*<0.60; *F*_Links_ = 2.26, *p*<0.16; *F*_Connectance_ = 0.27, *p*<0.62; Fig. 1). Annual number of butterfly species, however, increased slightly (0.9 butterfly species/yr; *F*_Animals_ = 5.27, *p*<0.043, Fig. 1). These results tell us that the data did not show any long-term trends. However, the annual estimates of *A*, *P*, *I* and *C* fluctuated. These patterns were measured as their signal-to-noise value 1/coefficient of variation (1/CV), which for the four parameters ranged from 0.07–0.11. Thus the global structure of the network demonstrated long-term temporal stability, but with annual fluctuations, especially in our estimates of *I*.

**Figure 2:**
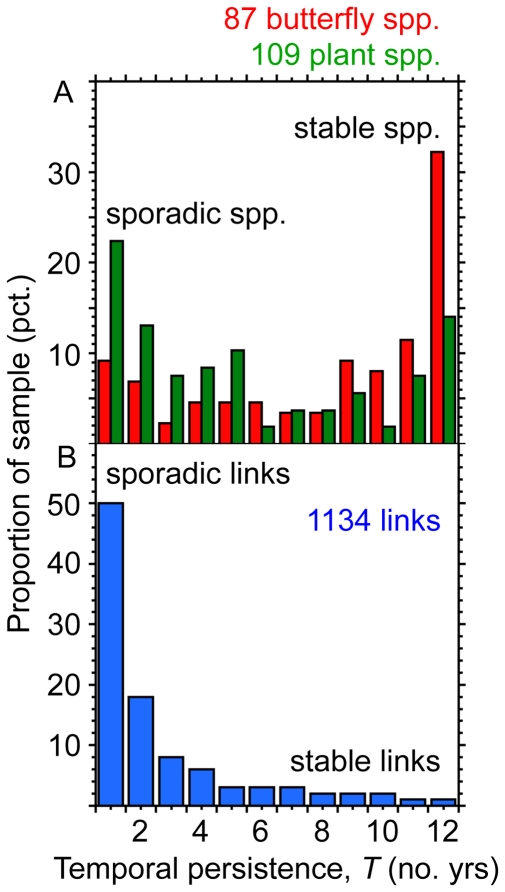
Variation in temporal persistence of species and links. Frequency distributions of temporal persistence *T* (no yrs observed) for species (*A*) and links (*B*). Sporadic species and links have a *T* of 1–2(-5) years, i.e. observed <20% of the time, whereas stable species and links have a *T* of (9-)11–12 years, i.e. observed >80% of the time.

In order to describe level of local stability in the network, we first calculated temporal persistence, *T*, of a species, i.e. the number of years it was present in the network. *T* was 8.2±4.0 yrs for butterflies, but only 5.5±4.1 yrs for plants, i.e. 68% and 46% of the 12 yr–study period, respectively (*T*_Butterflies_>*T*_Plants_: Wilcoxon rank sum test, *z* = 4.3, *p*<0.0001). Frequency distributions of *T* was bi-modally shaped (test, see [21]: an exact probability method comparing the probabilities of getting the leftmost and rightmost classes (and more extremes) with probabilities from a null model: *p*<0.05; Fig. 2*A*): one mode consisting of temporally stable species observed for 9–12 and 11–12 years for butterflies and plants, i.e. 60% and 21% of all butterfly and plant species, respectively; and another mode of temporally sporadic species observed only for 1–2 and 1–5 years, i.e. 16% and 61% of all butterfly and plant species, respectively. In contrast to species, links had a 1-modal and very skewed frequency distribution (mean *T* = 2.7±2.6 yrs, i.e. only 23% of the study period, Fig. 2*B*). As many as 68% of all links were sporadic, i.e. lasted only 1–2 yrs, and only 2% of all links were stable, i.e. lasted 11–12 yrs. Thus even among stable species, links were dynamical.

**Figure 3:**
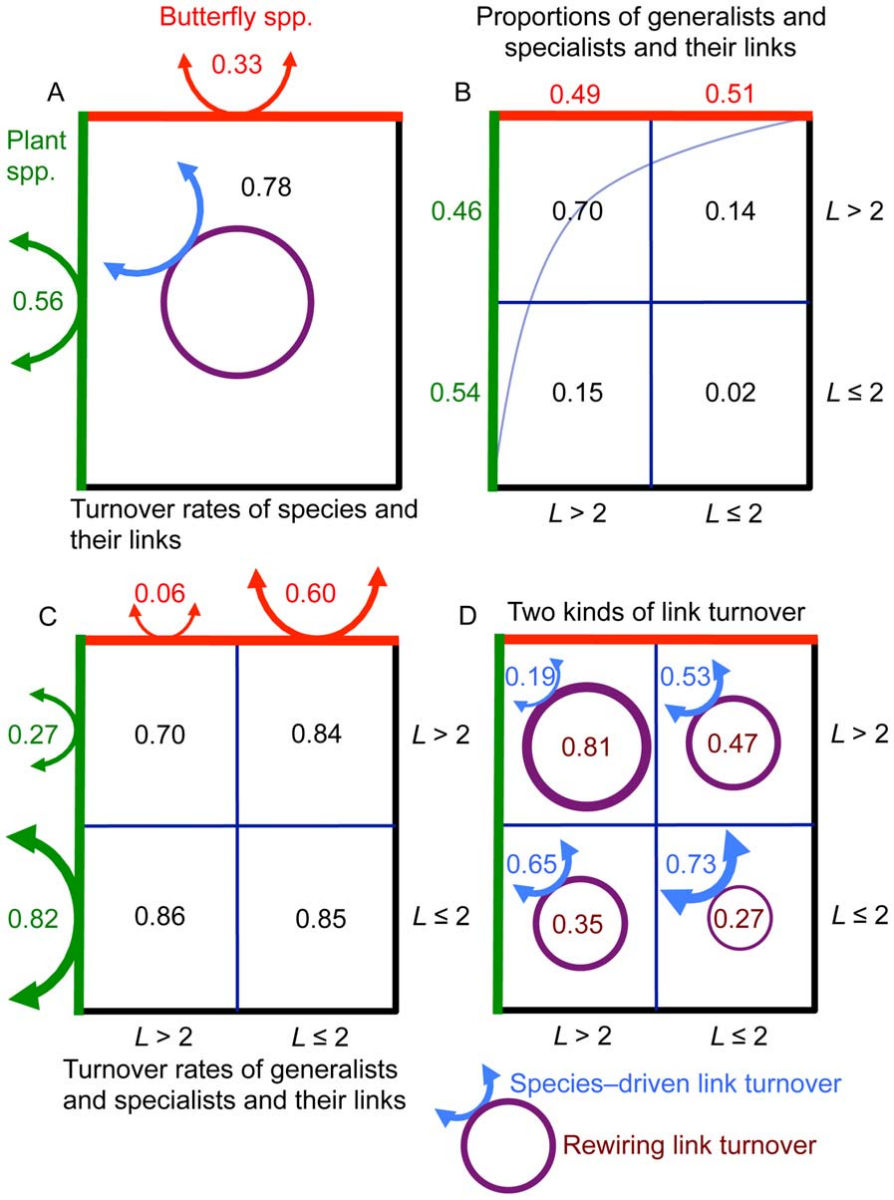
Numbers and annual turnover rates of species and links. Nested matrix between interacting butterfly species and their nectar plant species. The matrix is divided into quadrants, according to *L*>2 (generalists) or *L*≤2 links (specialists). (*A*) Overall average annual turnover rates for species and their links. Proportions of plant (green double arrow) and butterfly species turnover (red double arrow). Proportions of total link turnover attributed to rewiring (mauve circle) and species turnover (blue double arrow). (*B*) Proportions of generalist and specialist plant (green figures) and butterfly species (red figures) and their links in the 12-yr pooled matrix. The curved line is the nestedness isocline. (*C*) Average annual turnover rates for generalist and specialist plants (green double arrows) and butterfly species (red double arrows) and their links. (*D*) Proportions of total link turnover attributed to rewiring (mauve circles) and species turnover (blue double arrows).

In Fig. 3, species were sorted in a nested way according to their average annual linkage level *L*, i.e. their number of links to other species. All 12 annual matrices were significantly nested and the nestedness index *NODF* was stable across years (regression: *F*_1.11_ = 0.052, *p*<0.82). We termed species with an *L*>2 and *L*≤2 links for generalists and specialists, respectively. These two species groups were almost equally well represented in the network (Fig. 3*B*). However, 70% of all links connected generalists and only 2% connected specialists (Fig. 3*B*).

The low temporal persistence or *T*-value of many species and links indicated that their annual turnover was high. Indeed, more than half of all plant species, one third of all butterflies, and ¾ of all links disappeared or appeared in the network from year to year (Fig. 3*A*). Thus in our flower-visitation network, global stability and local instability co–occurred.

We went a step further in our analysis of the long–term dynamics of species and links by relating this turnover to their topological position in the network and we found a marked and complex temporal heterogeneity. Turnover *t* of specialists (*L*≤2 links) was 0.82 and 0.60 for plants and butterflies, respectively (Fig. 3*C*). Generalists (*L*>2 links), on the other hand, had a much lower *t*, viz. 0.27 and 0.06 for plants and butterflies, respectively (Fig. 3*C*). Species with an *L*>7 links had no turnover at all.

The low *t* for plant and butterfly generalists did not translate into a low *t* for their links (Fig. 3*C*). The latter was as high as 0.70, although not as high as *t* for the tail links, i.e. the links between generalists and specialists (0.84–0.86; comparison between *t* for generalists and specialists for the 11 annual transitions; Wilcoxon Rank sums: *z* = 7.41, *p*<0.0001). Links may go through two kinds of turnover, viz. rewiring and species turnover. In the first, “internal” links are rewired among species, which do not show any turnover, i.e. species already present in the network stay but they get new links, whereas in the latter, link turnover is driven by species turnover, i.e. new links build up as new species appear, and old links disappear together with their species. Thus we expected link rewiring to be most important in the core of generalists and species-driven link turnover to dominate in the tails. This was confirmed. In the core, 81% of total link turnover was due to link rewiring and only 19% was driven by species turnover, i.e. link rewiring was four times as high as species-driven link turnover. In the tails, on the other hand, species-driven link turnover was 1.1–1.9 times (0.53/0.47 = 1.1 to 0.65/0.35 = 1.9, Fig. 3*D*) as high as rewiring.

**Figure 4:**
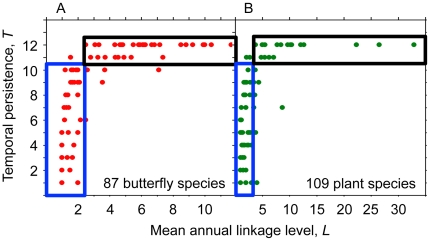
Relationship between temporal persistence of species and their linkage level. Relationship between temporal persistence *T* (no yrs observed) of butterfly species (*A*) and plant species (*B*) and their mean annual linkage level *L*. Two non-overlapping regions with respect to *T* and *L* were distinguished: The blue frame areas are regions of specialists, varying a lot in their *T*, and the black ones are regions of stable species, varying a lot in their *L*.

In the network, *L* increased with temporal persistence *T* (Fig. 4). In the *LT*-parameter space, species were restricted to two regions: a temporally dynamically “hot” region of specialists (*L*≤2 links), varying a lot in their *T*, albeit most *T*-values were low, and a temporally dynamical “cold” region of stable species (*T*≥11 yrs), varying a lot in their *L*, although all were generalists (Fig. 4). The plant *Eupatorium cannabarinum* was an outlier (*L* = 8 links and *T* = 7 yrs) (Fig. 4*B*). It invaded the network in the 6^th^ study year and as a fast-growing, clonal species with a large floral display it immediately achieved a high *L*. In the coming years, it will move up into the upper right corner of stable generalists (Fig. 4*B*). This region was made up of a few stable, extreme generalists belonging to the matrix core (6% and 2% of all butterfly and plant species, respectively).

Butterflies are, in general, regarded as generalized nectar-feeders, switching between flowers as they become available during their flight periods [22], and a longer adult phenophase was the most important single determinant increasing *L* (SI 1). Higher population abundance of butterflies, estimated independently of their flower visitation, also increased *L* (SI 1). Body size, measured as wing length, and habitat specialization both seemed to be unimportant to *L* (SI 1). In addition, *L* of a butterfly was positively correlated with the probability of encountering the same species at the other study sites (SI 1). If macroecology meets network analysis, we find that, generalist butterfly species at our study had a slightly larger (European) geographic range than specialists (SI 1). Finally, the set of generalist and specialist butterflies at the study site did not differ taxonomically at family level (SI 1), although Nymphalidae and Lycaenidae were slightly more common among specialists and Pieridae more so among generalists. In conclusion, adult phenophase and abundance, and geographic range influence *L* and thus the temporal dynamics of the network.

## Discussion

In our study, we did not include other visitors than butterflies. However, the temporal dynamics of butterfly-nectar plant networks is, without doubt, influenced by other flower-visiting insect orders. In general, Lepidoptera species only constitute an average (±SD) of 15% (±12%, range 3–44%) of the total visitor fauna in a network (sample: 56 networks; K. Trøjelsgaard, *pers. com.*), but a comparison of the interaction pattern of different flower-visiting insect orders has never been done.

The nectar plant-butterfly network showed both long-term global stability and local instability, i.e. over the study period of 12 years. A dynamical tension between the two levels has previously been reported from other networks [11]–[13]. We mapped this local instability in detail. The network was dominated by two distinct subsets of species, viz. sporadic species of low temporal persistence and stable species of high temporal persistence, and this created not just a strong local dynamics, but also a very asynchronous dynamics among these two kinds of species. This has also been reported for other communities, e.g. prairie herbs and estuarine fish [23], [24]. Temporal persistence *T* of a species was related to its linkage level *L*. Stable/high-*T* species varied in their *L*, although all were generalists (*L*>2 links). Specialists (*L*≤2 links) varied in their *T*, although most were sporadic (*T* = 1 to 2 yrs). In our network, two kinds of specialists are in action: “Evolutionary” specialists are species with a high *T* showing strong and temporally constant preferences for the same plant species, e.g. *Satyrium acaciae*, which across years mainly visited *Achillea millefolium* and *Anthemis triumfetti*, and “ecological” specialists with a low *T*, i.e. they became specialists in our notation because they were rare.

Annual turnover of species affected more than 1/3 of all species and ¾ of all links and was 3–10 times as high for specialists as for generalists. The most generalized species (*L*>7 links) were completely stable among years. In a system of pools and their invertebrates followed over 12 years, extinction and colonization were significantly correlated with species nestedness rank, equivalent to our *L* [25]. High-ranked species had lower extinction rate but higher colonization rate than lower ranked species. As in our study, extinction risk decreased with *L*, whereas colonization behaved oppositely, contrary to our results. Burgos *et al.* (2007) [26] demonstrated that if specialists have a higher risk of going extinct than generalists, as shown in our network, then a nested link pattern produces the most robust network. In the latter paper, robustness was defined as the cascading effect of an extinction of a species of one community onto the other, interacting community.

In our network, link turnover took place through two processes: Rewiring and species-driven turnover. The former was four times as common among the stable core species, whereas species-driven turnover dominated among the sporadic species. Thus two pronounced dynamics were observed in the network: (*i*) a strong temporal turnover of specialists and their links in the tails of the nested link pattern, and (*ii*) a strong temporal link turnover via rewiring among the temporally stable core of generalists. These two kinds of dynamics are driven by variation in *L* and species abundance and phenology. Behind (*i*) may be a metapopulational dynamical pattern, i.e. of specialists as transients failing to establish permanently [27]. Conclusion (*ii*) may be caused by high functional link redundancy among generalist species. Thus generalists are common and widespread species using the same set of common nectar plants seemingly without any preference. However, this needs to be tested in detail.

At present, we do not know whether this strong long-term dynamical picture observed in the plant-butterfly flower-visitation network may be generalized to other networks within and outside biology. However, the observed tension between global long-term stability and local instability is quite paradoxical and demonstrates the value of a dual research approach in future network analysis, i.e. a focus upon the network as well as the individual nodes.

## Supporting Information

SI1 **Linkage level and ecological correlates.**
(DOCX)
