# Supplementary material for: Strong, Long-Term Temporal Dynamics of an Ecological Network
Source: PLoS One. 2011 Nov 17;6(11):e26455. doi: 10.1371/journal.pone.0026455 (PMC3219636; doi:10.1371/journal.pone.0026455)
Supplement: SI1 — Linkage level and ecological correlates. (DOCX) [file pone.0026455.s001.docx]

In our study, we found that linkage level *L* was an important descriptor of species and link turnover. As an extension of this result, we analyzed a set of potential explanatory variables of *L*. We regressed *L* against these variables, using a phylogenetically controlled GLM analysis. All data used were from our study site El Puig.

Adult phenology is number of weeks each butterfly species is present at the study site.

*IA* is index of butterfly species abundance.

*SSI* is an index of habitat specialization.

Wing length is used as a proxy for body size.

Voltinism gives the number of generations per year.

Polyphagy gives the number of larval food plant species.

Model: linkage level *L* ~ Adult phenology + *IA* + *SSI* + Wing length + Voltinism +

Polyphagy + Intercept

Number of observations 87

Model:

Coefficients Estimate S.E. *t* *p*(>|*t*|)

Intercept 2.69 0.3508 7.66 0.000019

Adult phenology 0.0667 0.008967 7.44 0.000024

*IA* 0.000697 0.0001795 3.88 0.0031

*SSI* ‐0.0482 0.05585 ‐0.863 0.41

Wing length ‐0.00747 0.008557 ‐0.873 0.40

Voltinism ‐0.229 0.07516 ‐3.05 0.012

Polyphagy 0.0885 0.07280 1.22 0.25

Adult phenology and abundance (*IA*) were the most important predictors; voltinism

less so and the others were non-significant.

**Additional results used in the discussion:**

We made the same data sampling at three other study sites nearby:

Closes del Tec: UTM: X, Y: 509629, 4680432,

Can Liro: UTM: X, Y: 448922, 4615799, and

El Cortalet: UTM: X, Y: = 507851, 4674362,

- and found that

a) *L* was positively correlated with the probability of encountering the same species at the other study sites (*F*_1,85_ = 60.2, *p* < 0.001).

b) Generalist butterfly species had a slightly larger (European) geographic range (*t* =

2.01, *p* < 0.05) than specialists.

c) The set of generalist (*L*> 2 links) and specialist butterflies (*L* ≤ 2 links) at the study

site did not differ taxonomically at family level (*t*-paired = 0.87, *p* < 0.27).
